# A process evaluation of the NIDUS-Professional dementia training intervention for UK homecare workers

**DOI:** 10.1093/ageing/afae109

**Published:** 2024-05-25

**Authors:** Daniel Kelleher, Karen Windle, Rebecca Randell, Kathryn Lord, Larisa Duffy, Amirah Akhtar, Jessica Budgett, Sedigheh Zabihi, Sara Banks, Penny Rapaport, Teresa Lee, Julie Barber, Vasiliki Orgeta, Jill Manthorpe, Kate Walters, Kenneth Rockwood, Briony Dow, Juanita Hoe, Sube Banerjee, Claudia Cooper

**Affiliations:** Centre for Applied Dementia Studies, Faculty of Health Studies, University of Bradford, Richmond Road, Bradford, BD7 1DP, UK; Centre for Applied Dementia Studies, Faculty of Health Studies, University of Bradford, Richmond Road, Bradford, BD7 1DP, UK; Faculty of Health Studies, University of Bradford, Richmond Road, Bradford, BD7 1DP, UK; Centre for Applied Dementia Studies, Faculty of Health Studies, University of Bradford, Richmond Road, Bradford, BD7 1DP, UK; Division of Psychiatry, University College London, London, UK; Centre for Applied Dementia Studies, Faculty of Health Studies, University of Bradford, Richmond Road, Bradford, BD7 1DP, UK; Centre for Psychiatry and Mental Health, Wolfson Institute of Population Health, Queen Mary University London, London, UK; Centre for Psychiatry and Mental Health, Wolfson Institute of Population Health, Queen Mary University London, London, UK; Centre for Psychiatry and Mental Health, Wolfson Institute of Population Health, Queen Mary University London, London, UK; Division of Psychiatry, University College London, London, UK; Department of Statistical Science, University College London, London, UK; Department of Statistical Science, University College London, London, UK; Division of Psychiatry, University College London, London, UK; The Policy Institute at King’s, King’s College London, London, UK; Research Department of Primary Care and Population Health, University College London, London, UK; Division of Geriatric Medicine, Dalhousie University, Halifax, NS, Canada; National Ageing Research Institute, Melbourne, VIC, Australia; Geller Institute of Ageing and Memory, School of Biomedical Sciences, University of West London, London, UK; Faculty of Medicine and Health Sciences, University of Nottingham, Nottingham, UK; Centre for Psychiatry and Mental Health, Wolfson Institute of Population Health, Queen Mary University London, London, UK

**Keywords:** dementia, homecare, training, carers, process evaluation, implementation, older people

## Abstract

**Introduction:**

This process evaluation was conducted in parallel to the randomised controlled feasibility trial of NIDUS-Professional, a manualised remote dementia training intervention for homecare workers (HCWs), delivered alongside an individualised intervention for clients living with dementia and their family carers (NIDUS-Family). The process evaluation reports on: (i) intervention reach, dose and fidelity; (ii) contexts influencing agency engagement and (iii) alignment of findings with theoretical assumptions about how the intervention might produce change.

**Methods:**

We report proportions of eligible HCWs receiving any intervention (reach), number of sessions attended (dose; attending ≥4/6 main sessions was predefined as adhering), intervention fidelity and adherence of clients and carers to NIDUS-Family (attending all 6–8 planned sessions). We interviewed HCWs, managers, family carers and facilitators. We integrated and thematically analysed, at the homecare agency level, qualitative interview and intervention recording data.

**Results:**

32/141 (23%) of eligible HCWs and 7/42 (17%) of family carers received any intervention; most who did adhered to the intervention (89% and 71%). Intervention fidelity was high. We analysed interviews with 20/44 HCWs, 3/4 managers and 3/7 family carers, as well as intervention recordings involving 32/44 HCWs. All agencies reported structural challenges in supporting intervention delivery. Agencies with greater management buy-in had higher dose and reach. HCWs valued NIDUS-Professional for enabling group reflection and peer support, providing practical, actionable care strategies and increasing their confidence as practitioners.

**Conclusion:**

NIDUS-Professional was valued by HCWs. Agency management, culture and priorities were key barriers to implementation; we discuss how to address these in a future trial.

## Key Points

- Homecare workers (HCWs) valued the rare opportunity to speak with peers, reflect on experiences and learn new strategies.
- Training increased HCW skills and confidence, empowering practice change.
- The intervention was delivered with high fidelity and achieved high adherence among those who received it.
- Staff shortages, heavy workloads, competing priorities and management buy-in were key barriers to implementation.
- Flexibility, including remote delivery and individual catch-up sessions, helped increase the intervention ‘dose’.

## Introduction

An estimated 400,000 UK people living with dementia and their families rely on paid care services [1]. Additional dementia-specific training for homecare workers (HCWs) is needed [2]. Interventions have improved the quality of care for those living with dementia in care homes [3, 4], but HCWs often work alone in clients’ homes, so their role and training needs differ from those of staff working in communal settings. Evidence is limited on how to develop sustainable training models in a setting characterised by staff turnover, heavy workloads and tight schedules [5].

We conducted a feasibility randomised controlled trial of NIDUS-Professional, a training and support intervention for HCWs that aimed to improve staff sense of competence in dementia care, reduce burnout and improve the quality of care and life of clients with dementia. NIDUS-Professional (Supplementary Table S1; supplementary material), previously reported to have broadly met criteria for progression to a full trial [6], comprised six, 1–1.5 hour, manualised video-call group sessions delivered over 3 months by two non-clinical facilitators, then 3 monthly catch-up groups to support application of learning into practice. HCWs who could not attend a session received individual catch-up sessions. Agency managers were offered three individual sessions. Eligible clients and carers were offered the dyadic NIDUS-Family intervention, described elsewhere [7–9].

**Figure 1:**
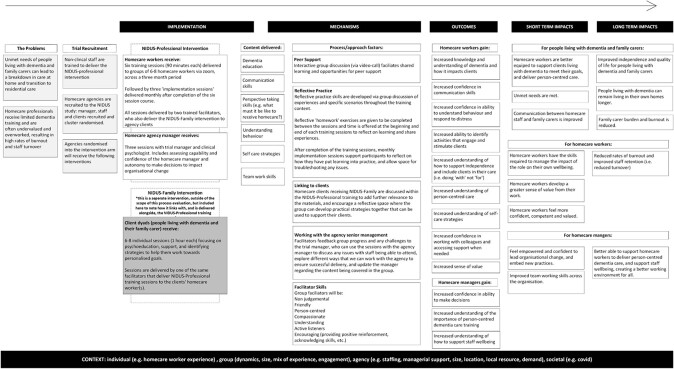
Logic model.

Process evaluations are increasingly used alongside complex intervention trials [10–13]; this process evaluation aimed to contextualise our findings, informing the future trial. Our NIDUS-Professional logic model (Fig. 1) outlines theoretical assumptions about how the intervention might work, and guided by this model, we aimed to report: (aim 1) intervention reach, dose and fidelity; (aim 2) contexts influencing agency engagement and (aim 3) how findings align with theoretical assumptions about intervention mechanisms.

## Methods

### Study design

To understand how context influenced NIDUS-Professional implementation [14], we used the MRC guidance on evaluating complex interventions [13]. The design and development of the logic model (see Figure 1) demanded a theory-driven, mixed methods and case study design, where cases were intervention arm agencies. The study was approved by London-Camden and King’s Cross National Research Ethics Committee (20/LO/0567); registered protocol ISRCTN15757555.

### Data collection

Following informed consent, socio-demographic details were collected within the NIDUS-Professional RCT, with attendance data logged at training and follow-up sessions [10]. All HCWs were invited to participate in focus groups and/or interviews following completion of the main sessions (at 3 months) (semi-structured interview guide, Appendix S1) and for feedback regarding monthly catch-up sessions via email or phone at 6 months (Appendix S2). Participants not interviewed at 3 months were invited for interviews at 6 months. Managers were interviewed separately. Interviews were conducted with carers to explore potential links between the NIDUS-Professional and NIDUS-Family interventions. We invited intervention facilitators, the supervising clinical psychologist and the trial manager to individual interviews. All data collection was done remotely, by telephone, video-call or email, due to pandemic restrictions. Interviews and intervention sessions were audio-recorded.

### Analysis

We used recruitment and attendance logs to calculate reach: the proportion of participants referred to the study from intervention arm agencies receiving intervention sessions. Dose was calculated as the proportion of planned intervention sessions completed. D.K. and A.A. (NIDUS researchers) independently listened to audio recordings of two randomly selected interventions per group, completing fidelity checklists. We calculated the proportion of expected intervention components (Supplementary Table S1; supplementary material) delivered. In applying the observational Fidelity Checklist (developed for this study and not validated) [6], we rated fidelity using established thresholds, 81%–100% constituting high fidelity. Using a 5-point scale (1- not at all to 5-very much), we rated whether facilitators kept the group focused on the manual and participants engaged for each intervention component, and whether the session kept to time. We calculated summary descriptive statistics for fidelity using Excel.

### Qualitative analysis

Audio recordings of interviews, focus groups and intervention sessions were transcribed, and email feedback data was extracted and analysed in NVivo12. A thematic analysis was applied [15]. D.K. (a NIDUS researcher with a psychology background) developed an initial coding framework from the logic model. He deductively coded textual data for one focus group and interview, then inductively coded the transcripts into meaningful fragments to identify themes not captured by the deductive framework. K.W. and R.R. (D.K.’s PhD supervisors) independently assessed the coding framework alongside the data, refining as necessary. D.K., K.W. and R.R. agreed on the final coding frame, which D.K. applied to all data. A matrix was developed to identify themes within each case study (agency). D.K., K.W. and R.R. discussed the developing themes on four more occasions, discussing inconsistencies to refine them [14, 15].

### Reflexivity

We drew on reflexive practices during analysis, considering how our subjectivity and interpersonal, including power dynamics between researchers and participants, influenced our findings. Data were analysed before the trial outcome [6] was known. Neither D.K., K.W. nor R.R. were involved in the trial or intervention delivery, so they had a relative outsider stance. This independence may not have eliminated a desirability bias in participants when reporting their experiences of the intervention or the influence of insider perspectives within the co-author team. Other co-authors, C.C. (Chief Investigator) and L.D./J.Bu. (NIDUS-Professional and NIDUS-Family trial managers), had an insider stance. We were conscious of our positionality, as insiders, in relation to the NIDUS study and discussed this, giving weight to the perspectives of K.W. and R.R. who were outside this research group.

## Results

### Setting and sample description

**Figure 2:**
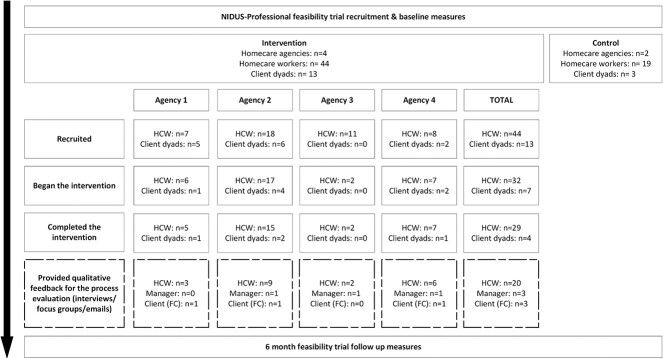
Flowchart mapping the relationship between the process evaluation sample and the feasibility trial population.

D.K. interviewed 20/44 HCWs (13 HCWs across 2 focus groups, 7 individual interviews), 3/4 managers (all women) and 3/7 carers (all clients’ sons), all of whom participated in the intervention (Supplementary Table 1). We included audio-recording transcripts from 28/30 HCW group sessions (NIDUS-Professional was delivered in three parallel groups in agency 2 but in agency 3 only individual catch-up sessions were possible (5/11 recorded)); 32/44 HCWs participated in at least one recorded session, including 12 HCWs who were not interviewed. We interviewed three facilitators (all women), the supervising clinical psychologist and the trial manager. The sociodemographic characteristics of HCWs participating in the process evaluation are described in Table 2 and compared to the whole intervention arm population. Figure 2 compares the process evaluation sample to the feasibility trial population.

**Table 1:** Process evaluation qualitative data

	Case study data			
	Agency 1	Agency 2	Agency 3	Agency 4
**HCWs**				
Focus groups	0	1 (*n* = 7)	0	1 (*n* = 5)
Interviews	3	2	1	0
Email feedback	3	4	1	3
**Managers**				
Interviews	0	1	1	0
Email feedback	0	0	0	1
**Family carers**				
Interviews	1	1	0	1
**Transcribed intervention audio recordings**				
HCW sessions	6	19	5	6
FC sessions	5	0	0	5
	**General trial feedback data**			
**Researchers and facilitators**				
Facilitator interviews	3			
Clinical supervisor interview	1			
Trial manager interview	1			

**Table 2:** Homecare worker characteristics in the process evaluation sample and trial population, within the intervention arm

	Agency 1		Agency 2		Agency 3		Agency 4		Total intervention population (*n* = 44)
Results are *n*/*N* (%) unless specified otherwise	PE (*n* = 3)	Trial (*n* = 7)	PE (*n* = 9)	Trial (*n* = 18)	PE (*n* = 2)	Trial (*n* = 11)	PE (*n* = 6)	Trial (*n* = 8)	
**Age (yr), median (LQ,UQ)**	56.5 (54.8, 62.7)	56.8 (54.8, 63.7)	38.2 (30.5, 48.3)	40.2 (32.5, 52.2)	43.5 (32.7, 54.3)	48 (43.9, 54.3)	52.6 (36/6. 57.1)	53.5 (43.6, 61.6)	50.8 (38.8, 56.6)
**Gender**									
Women	3/3 (100)	7/7 (100)	9/9 (100)	18/18 (100)	2/2 (100)	11/11 (100)	6/6 (100)	8/8 (100)	44/44 (100)
**Ethnicity**									
White British	3/3 (100)	7/7 (100)	8/9 (88.9)	16/18 (88.9)	2/2 (100)	11/11 (100)	3/6 (50)	5/8 (62.5)	39/44 (88.6)
Other	0/3 (0)	0/7 (0)	1/9 (11.1)	2/18 (11.1)	0/2 (0)	0/11 (0)	3/6 (50)	3/8 (37.5)	5/44 (11.4)
**First language**									
English	3/3 (100)	7/7 (100)	8/9 (88.9)	16/18 (88.9)	2/2 (100)	11/11 (100)	3/6 (50)	5/8 (62.5)	39/44 (88.6)
Other	0/3 (0)	0/7 (0)	1/9 (11.1)	2/18 (11.1)	0/2 (0)	0/11 (0)	3/6 (50)	3/8 (37.5)	5/44 (11.4)
**Highest level of education**									
Degree or higher	1/3 (33.3)	3/7 (42.9)	1/9 (11.1)	2/18 (11.1)	0/2 (0)	1/11 (9.1)	2/6 (33.3)	2/8 (25)	8/44 (18.2)
Vocational	1/3 (33.3)	2/7 (28.6)	3/9 (33.3)	9/18 (50)	2/2 (100)	8/11 (72.7)	3/6 (50)	4/8 (50)	23/44 (52.3)
Secondary school/college	0/3 (0)	0/7 (0)	5/9 (55.6)	7/18 (38.9)	0/2 (0)	2/11 (18.2)	0/6 (0)	0/8 (0)	9/44 (20.5)
No formal qualification	1/3 (33.3)	1/7 (14.3)	0/9 (0)	0/18 (0)	0/2 (0)	0/11 (0)	0/6 (0)	0/8 (0)	1/44 (2.3)
Other	0/3 (0)	1/7 (14.3)	0/9 (0)	0/18 (0)	0/2 (0)	0/11 (0)	1/6 (16.7)	2/8 (25)	3/44 (6.8)
**Dementia training**									
Yes	2/3 (66.7)	6/7 (85.7)	7/9 (77.8)	15/18 (83.3)	2/2 (100)	11/11 (100)	4/6 (66.7)	6/8 (75)	38/44 (86.4)
No	1/3 (33.3)	1/7 (14.3)	2/9 (22.2)	3/18 (16.7)	0/2 (0)	0/11 (0)	2/6 (33.3)	2/8 (25)	6/44 (13.6)
**Days of training**									
1 d or less	2/2 (100)	4/5 (80)	4/7 (57.1)	6/15 (40)	2/2 (100)	7/11 (63.6)	3/4 (75)	5/6 (83.3)	22/37 (59.5)
2–3 d	0/2 (0)	1/5 (20)	1/7 (14.3)	2/15 (13.3)	0/2 (0)	1/11 (9.1)	0/4 (0)	0/6 (0)	4/37 (10.8)
4 or more	0/2 (0)	0/5 (0)	2/7 (28.6)	7/15 (46.7.9)	0/2 (0)	3/11 (27.3)	1/4 (25)	1/6 (16.7)	11/37 (29.7)
Missing		*1*							*1*
**Employment**									
Homecare worker	3/3 (100)	6/7 (85.7)	9/9 (100)	17/18 (94.4)	2/2 (100)	6/11 (54.5)	6/6 (100)	7/8 (87.5)	36/44 (81.8)
Homecare manager	0/3 (0)	0/7 (0)	0/9 (0)	0/18 (0)	0/2 (0)	1/11 (9.1)	0/6 (0)	0/8 (0)	1/44 (2.3)
Other	0/3 (0)	1/7 (14.3)	0/9 (0)	1/18 (5.6)	0/2 (0)	4/11 (36.4)	0/6 (0)	1/8 (12.5)	7/44 (15.9)
**Working hours**									
Full-time	1/3 (33.3)	2/7 (28.6)	3/9 (33.3)	9/18 (50)	1/2 (50)	4/11 (36.4)	4/6 (66.7)	6/8 (75)	21/44 (47.7)
Part-time	2/3 (66.7)	5/7 (71.4)	5/9 (55.6)	7/18 (38.9)	1/2 (50)	5/11 (45.5)	2/6 (33.3)	2/8 (25)	19/44 (43.2)
Other		0/7 (0)	1/9 (11.1)	2/18 (11.1)	0/2 (0)	2/11 (18.2)	0/6 (0)	0/8 (0)	4/44 (9.1)
**Time worked in current agency**									
Less than 6 mo	0/3 (0)	0/7 (0)	1/9 (11.1)	1/18 (5.6)	0/2 (0)	1/11 (9.1)	0/6 (0)	0/8 (0)	2/44 (4.6)
6 mo to 1 yr	0/3 (0)	1/7 (14.3)	1/9 (11.1)	2/18 (11.1)	0/2 (0)	2/11 (18.2)	1/6 (16.7)	1/8 (12.5)	6/44 (13.6)
1–3 yr	2/3 (66.7)	2/7 (28.6)	3/9 (33.3)	5/18 (27.8)	1/2 (50)	2/11 (18.2)	4/6 (66.7)	5/8 (62.5)	14/44 (31.8)
3–5 yr	1/3 (33.3)	3/7 (42.9)	0/9 (0)	2/18 (11.1)	0/2 (0)	2/11 (18.2)	1/6 (16.7)	2/8 (25)	9/44 (20.5)
5–10 yr	0/3 (0)	1/7 (14.3)	3/9 (33.3)	4/18 (22.2)	1/2 (50)	4/11 (36.4)	0/6 (0)	0/8 (0)	9/44 (20.5)
10+ yr	0/3 (0)	0/7 (0)	1/9 (11.1)	4/18 (22.2)	0/2 (0)	0/11 (0)	0/6 (0)	0/8 (0)	4/44 (9.1)
**Time worked in homecare overall**									
Less than 6 mo	0/3 (0)	0/7 (0)	1/9 (11.1)	1/18 (5.6)	0/2 (0)	1/11 (9.1)	0/6 (0)	0/8 (0)	2/44 (4.6)
6 months to 1 yr	0/3 (0)	1/7 (14.3)	1/9 (11.1)	1/18 (5.6)	0/2 (0)	0/11 (0)	1/6 (16.7)	1/8 (12.5)	3/44 (6.8)
1–3 yr	2/3 (66.7)	2/7 (28.6)	2/9 (22.2)	5/18 (27.8)	0/2 (0)	0/11 (0)	2/6 (33.3)	2/8 (25)	9/44 (20.5)
3–5 yr	0/3 (0)	1/7 (14.3)	0/9 (0)	2/18 (11.1)	0/2 (0)	1/11 (9.1)	1/6 (16.7)	1/8 (12.5)	5/44 (11.4)
5–10 yr	0/3 (0)	1/7 (14.3)	3/9 (33.3)	3/18 (16.7)	0/2 (0)	4/11 (36.4)	2/6 (33.3)	2/8 (25)	10/44 (22.7)
10+ yr	1/3 (33.3)	1/7 (14.3)	2/9 (22.2)	6/18 (33.3)	2/2 (100)	5/11 (45.5)	0/6 (0)	2/8 (25)	14/44 (31.8)
Unable to specify	0/3 (0)	1/7 (14.3)	0/9 (0)	0/18 (0)	0/2 (0)	0/11 (0)	0/6 (0)	0/8 (0)	1/44 (2.3)

PE*,* process evaluation; LQ, lower quartile; UQ, upper quartile.

### Reach, dose and fidelity (aim 1)

Table 3 summarises reach and dose.

**Table 3:** Homecare worker and client dyad recruitment, intervention delivery, reach and dose in the trial intervention arm

		Number of participants						
Agency	Participant group	Referred	Randomised to intervention	Completed 1+ session	Completed intervention HCW 4/6, FC 6/8 sessions	Reach No. who started intervention/no. referred	Overall dose No. of sessions completed/no. of total sessions possible	Dose (in those attending 1+ session)
**1**	**HCW**	40	7	6	5	6/40 (15%)	31/42 (74%)	31/36 (86%)
	**FC**	20	5	1	1	1/20 (5%)	8/40 (20%)	8/8 (100%)
**2**	**HCW**	19	18	17	15	17/19 (89%)	88/108 (81%)	88/102 (86%)
	**FC**	9	6	4	2	4/9 (44%)	23/48 (48%)	23/32 (72%)
**3**	**HCW**	28	11	2	2	2/28 (7%)	11/66 (17%)	11/12 (92%)
	**FC**	4	0	0	0	0/4 (0%)	0/0 (0%)	0/0 (0%)
**4**	**HCW**	54	8	7	7	7/54 (13%)	40/48 (95%)	40/42 (95%)
	**FC**	9	2	2	1	2/9 (22%)	9/16 (56%)	9/16 (56%)
**Total**	**HCW**	141	44	32	29	32/141 (23%)	170/264 (64%)	170/192 (89%)
	**FC**	42	13	7	4	7/42 (17%)	40/104 (39%)	40/56 (71%)

**Reach:** Supplementary Tables S2 and S3 (supplementary material) show HCW and carer intervention adherence. 32/141 (23%) HCWs and 7/42 (17%) carers referred by intervention arm agencies received any intervention.

**Dose:** 29/44 (65.9%) HCWs randomised to the intervention completed at least four intervention sessions (reported previously [6]). 170/264 (64%) main HCW sessions were completed (170/192 (89%) among HCWs attending any sessions). 7/13 carers randomised to the intervention completed any NIDUS-Family sessions: 4/7 family carers completed the NIDUS-Family intervention (attending 6–8 sessions), 2 attended 4 sessions and 1 attended a single session. In total, 40/104 (39%) carer sessions were completed (40/56 (71%) among carers who attended any intervention sessions).

**Fidelity:** All 36 intervention session components recorded were delivered, indicating that fidelity (36/36: 100%) was high. Median rater fidelity scores were: 5 (interquartile range: 0) for ‘Keeping the group focused on the manual/task’; 5 (1) for ‘Keeping participants engaged’ and 5 (1) for ‘Keeping the session on time’. One agency manager attended all 3 manager sessions, 2 attended 2 sessions and received written feedback in lieu of the final session and 1 manager (Agency 3) attended no sessions.

### Qualitative findings

We report our qualitative findings in two sections, corresponding to our second and third aims (see introduction).

### Contexts influencing implementation (aim 2)

In exploring agency contexts, we identified one overarching theme: ‘implementation requires planning, flexibility and understanding of wide-ranging structural challenges’.

All agencies experienced challenges with intervention attendance. Agencies 1 and 3 managers asked HCWs to complete training outside working hours; in Agency 1, there were geographical challenges to arranging staff cover, while in Agency 3, where reach and dose were lowest, staffing issues meant that training could not be prioritised:

They said if you do it you have to do it in your own time…we’re so spaced out, it's not ‘can you cover so and so’ because it's, you’re just round the corner, it's not like that. (Agency1, HCW1, Interview).

We had some members of the team quit (unexpectedly)…quite a few carers were off with Covid, and we were trying to juggle annual leave (during school holidays) …the clients still needed to be seen so most of the office staff were also out delivering care, which left no time for anything else. (Agency3, Manager, Interview).

Managers in agencies 2 and 4 supported staff attendance, reflected in higher intervention dose and reach, but the practical challenges of creating space for training in an at-capacity system were evident; at times HCWs needed to prioritise it over breaks between clients or could not attend as they prioritised their clients:

It was a big job to free people up. We still had people doing it literally coming out of calls and sitting in their cars before they went to their next call, because that was the only time that they had. (Agency2, HCW2, Interview).

Some of the carers are quite attached to the customers, so they'll be like ‘actually no, I'd rather go and do my call thanks, and I'll do it another time.’ (Agency2, HCW2, Interview).

Aligning with our logic model, the option to complete individual or small group catch-up sessions helped mitigate these challenges. HCWs valued the remote training format, allowing them the flexibility to attend sessions from home, often in their personal time:

I wasn't able to attend a couple…so they were able to re-jig when I did those, which was great. (Agency1, HCW2, Interview).

I think it's a benefit being online because we are in the comfort of our own homes. (Agency4, HCW2, Focus Group).

Structural challenges, including staff turnover and a lack of continuity of care, limited our attempts to establish the intended links between NIDUS-Family and NIDUS-Professional:

To say, OK, let's discuss this client with this HCW and their carer and talk about the ways that we can all pitch in, it just doesn't work because they're constantly changing, and you don't know who's going in next week. (Facilitator1, Interview).

He did have somebody who would be visiting him on a very regular basis. She was taking part in the sessions and we were talking about what we discussed…she had cards, she brought in a small keyboard for him to have a play with, but unfortunately, she's not there anymore. (Agency4, FC, Interview).

Discussions of potential challenges, the agency’s readiness to receive the intervention, and how managers could help mitigate structural barriers were scheduled for one-to-one meetings with agency managers. One manager attended all sessions, but others were difficult to reach, and practical conversations were often prioritised over the intended discussions about training implementation and readiness for organisational change:

It felt as if the managers were coming at the conversation from let's just get the job done…instead of thinking more richly about what they’d stepped into, how they might take it forward. (NIDUS Clinical Psychologist, Interview).

One possible explanation for low attendance was that managers did not feel part of the training and reported that their roles felt more administrative. To Agency 3 manager, separation of manager and HCW sessions felt unacceptable:

My role was just as admin. It would’ve been helpful for me to have gone to the training. Whether I’d have had time is another thing, but I was told that I couldn't do it. (Agency3, Manager, Interview)

### Theoretical assumptions about how the intervention might produce change (aim 3)

We identified three themes, describing: (i) benefits of group reflection and peer support; (ii) how discussions produced practical, actionable strategies and (iii) how the intervention empowered HCWs generating ‘increased confidence in HCW practice’.

***Theme 1: Benefits of group reflection and peer support***

Consistent with our logic model (Figure 1), participants valued the rare opportunity to reflect on their practice and learn from others’ experiences. By positioning HCWs as the ‘experts’, facilitators promoted reflective group discussions, which offered reassurance for many that they were not alone in their struggles:

We just tend to have a day and then we go home…whereas to actually bring our mind into focus about dementia, how we feel, where do we get support from, do I feel relaxed, do I feel stressed? It's good to start asking those questions and reflect on it. (Agency2, HCW3, Session 1 Recording).

Having similar shared experiences, you know it's not just me experiencing, it's everybody else which is quite reassuring. (Agency1, HCW4, Session 1 Recording).

Participants in Agency 3, who received one-to-one sessions only, noted that this core component of peer-to-peer discussion would have been welcome:

It would be good to have heard from the other people at work. Like we really don’t see each other. (Agency3, HCW1, Interview).

*Theme 2: Discussions produced practical, actionable strategies*

All participants welcomed the training’s practical focus and reported applying learning to improve client care, including innovative communication, ideas for enjoyable activities, improved understanding of behaviour and relaxation exercises to alleviate clients’ anxieties:

Not only are we benefiting but primarily our people who we’re looking after are benefiting. It is just fabulous, and you can make even more difference to those people and their families with the support that you can offer if we’ve got some more strategies, like we’ve learned from talking to each other. (Agency4, HCW2, Focus Group).

I’ve been using some of the breathing exercises with a client. When using the hoist she could be upset sometimes but doing the breathing with her helps her be more calm. (Agency2, HCW8, Email).

HCWs also reported using strategies to promote their own wellbeing and developing new support systems:

I really enjoy the relaxation technique and if you are relaxed then you have a different kind of energy to take with you for work. (Agency4, HCW3, Session 5 Recording).

After the meetings I’ve started to meet up with another caregiver for coffee so we can discuss our problems with similar clients and pass on information about work which has been very helpful. (Agency1, HCW1, Interview).

*Theme 3: Increased confidence in HCW practice*

Participants reported increased confidence in their skills, with some feeling empowered to advocate for change. This included sharing learning with colleagues, asking management to implement improvements to agency systems and care planning processes, establishing peer support groups, and requesting additional dementia training for those unable to receive NIDUS-Professional:

We’re all writing more in the care notes about strategies we use and things we do with a customer that works so new carers coming in can get more information about the customer…more than just what needs doing. (Agency2, HCW5, Email).

I did mention to the owner that other care workers would definitely benefit from extra training regarding dementia and a training session was arranged…I also asked my manager if I can be shadowed by other staff so I can pass on information about that client and how they may react to different ways of doing things. (Agency1, HCW1, Email).

## Discussion

While only a quarter of HCWs in intervention agencies received any sessions, nearly 9 in 10 who engaged adhered fully to the intervention, which was delivered with high fidelity. Initial buy-in was the key barrier to achieving a higher intervention dose, influenced by agency management, culture and priorities. Aligning with our logic model and adult learning theory [16], HCWs valued the opportunity to speak with peers, reflect on their practice and learn new strategies. The sessions fostered new connections between HCWs and requests for agency-level peer support. Individual and agency-level goals and actions appeared to influence care planning and led to requests for access to more dementia training.

Our findings contribute to the evidence that where HCWs are supported to build skills, confidence and a sense of value in their work through peer support, reflective practice and practical strategies, they are enabled to deliver better quality care [5, 17, 18]. Implementation barriers included a lack of protected time and management support. Staff shortages, turnover and high demand for care meant that HCWs were often unable to attend sessions, despite being reimbursed for training time [6]. Flexibility, including remote delivery and individual catch-up sessions, helped increase intervention ‘dose’, though without the benefits of peer discussions.

Our findings align with research in care homes, showing that leadership style and how well managers understand, value and engage with an intervention are key to implementation [19]. Managers did not feel part of NIDUS-Professional. Strategies to engage more effectively with agency management in a future trial might include paying HCWs for time to champion and co-facilitate the intervention and reviewing how the manager is included in sessions with HCWs. HCW-only support groups were co-designed to provide space for HCWs to discuss agency challenges, but one manager told us she felt excluded, and this likely contributed to low adherence in that agency.

### Limitations

It proved challenging to test the linked delivery of the NIDUS interventions because of challenges in recruiting clients and high HCW turnover. Previous interventions recruited convenience samples, for example, the Australian Promoting Independence Through quality Care at Home (PITCH) RCT (reporting soon) [20]. We tried to recruit all HCWs and clients from participating agencies. While closer to real-world practice, this was challenging. Many relevant levers operate at sector rather than agency-level, e.g. regulatory requirements for training. Study limitations include biases in the agencies participating in the main trial, towards higher CQC-rated and inevitably more outward-facing providers, compounded by biases in the proportion of trial participants who took part in the process evaluation. No male HCWs participated in the trial. Most HCWs were White British with English as a first language, so our findings may be less applicable to more diverse communities. Our interview sample is biased towards those with greater engagement; 19/20 (95%) HCWs and 3/3 (100%) family carers interviewed completed the intervention. Through including recorded training sessions, we captured more voices; this may have introduced desirability bias as intervention facilitators were present, though data from session and post-intervention recordings was broadly aligned.

## Conclusion

The willingness of resource-stretched agencies and HCWs to engage with this video-call intervention is promising. Our feasibility trial met criteria to progress to a full trial, with adaptations informed by this process evaluation and feasibility trial findings, such as using aggregated, anonymised agency outcomes, collected by agency-employed champions, to circumvent the difficulties of recruiting clients, especially those without a family carer, as trial participants. If a pragmatic trial demonstrates effectiveness, NIDUS-Professional will enable the rollout of training in the sector, a current policy priority [20].

## Supplementary Material

aa-23-1839-File002_afae109
